# Why do some coronaviruses become pandemic threats when others do not?

**DOI:** 10.1371/journal.pbio.3001652

**Published:** 2022-05-16

**Authors:** Benjamin L. Rice, Justin Lessler, Clifton McKee, C. Jessica E. Metcalf

**Affiliations:** 1 Department of Ecology and Evolutionary Biology, Princeton University, Princeton, New Jersey, United States of America; 2 Department of Epidemiology, University of North Carolina, Chapel Hill, North Carolina, United States of America; 3 Department of Epidemiology, Bloomberg School of Public Health, Johns Hopkins University, Baltimore, Maryland, United States of America; 4 Princeton School of Public and International Affairs, Princeton University, Princeton, New Jersey, United States of America; 5 Wissenschaftskolleg zu Berlin, Berlin, Germany

## Abstract

Despite multiple spillover events and short chains of transmission on at least 4 continents, Middle East Respiratory Syndrome Coronavirus (MERS-CoV) has never triggered a pandemic. By contrast, its relative, Severe Acute Respiratory Syndrome Coronavirus 2 (SARS-CoV-2) has, despite apparently little, if any, previous circulation in humans. Resolving the unsolved mystery of the failure of MERS-CoV to trigger a pandemic could help inform how we understand the pandemic potential of pathogens, and probing it underscores a need for a more holistic understanding of the ways in which viral genetic changes scale up to population-level transmission.

## Abstract 2

What does it take for a coronavirus to become a pandemic threat? And why has MERS-CoV repeatedly failed to emerge as a pandemic pathogen when SARS-CoV-2 has successfully spread around the world?

## Introduction

In the past 2 decades, 3 coronaviruses have jumped from animals to successfully cause epidemics in the human population. The first, Severe Acute Respiratory Syndrome Coronavirus (SARS-CoV-1), emerged in 2002, causing more than 8,000 cases and hundreds of deaths, but was successfully contained [1]. The second, Middle East Respiratory Syndrome Coronavirus (MERS-CoV), was first identified in April 2012 and has continued to cause human infections, resulting in at least 2,000 cases originating from repeated spillover events [2]. Thought to predominantly come from contact with dromedary camels [3], infections have largely remained contained in the region around Saudi Arabia. The third, Severe Acute Respiratory Syndrome Coronavirus 2 (SARS-CoV-2), which emerged in 2019, is causing an ongoing pandemic, millions of deaths, and vast disruption to daily life. What has defined the different trajectories for these related viruses?

Given the potentially vast diversity of animal-infecting coronaviruses, and many opportunities for contact (direct or indirect) between humans and zoonotic hosts, there is reason to suspect that unrecorded zoonotic coronavirus infections of human hosts are frequent. Indeed, serological assays suggest frequent exposure in areas where contact with animals is likely [4–7]. The frequency of such exposures might therefore not be the rate limiting step for outbreaks with pandemic potential. However, details of the exposure event, including viral dose, host age and immune status [8,9], and opportunities for onward transmission from the exposed host, may all influence the prospects of an outbreak and subsequent sustained transmission in human populations. In addition, some viruses may enter the human population lacking the capacity for efficient spread between humans, in which case adaptation to humans after a spillover event could be the critical barrier. Alternatively, evolution in the pre-spillover host (i.e., the reservoir) could result in the acquisition of traits capable of sustaining human-to-human transmission (“preadaptation”) and thus this acquisition might prove the critical barrier.

For an emergent pathogen to sustain spread within the human population, on average, each infected individual must infect at least one other. Epidemiologists capture this requirement using a quantity denoted R, defined as the expected number of new infections per infected person. Estimating this quantity requires that not only the daily rate at which an infected individual transmits to others be accounted for, but also how long the individual remains infected before they recover, die, or otherwise lose the ability to transmit (e.g., are isolated from others).

Both SARS-CoV-1 and SARS-CoV-2 seem to have entered the human population with, or quickly acquired, the right combination of these features to spread widely. One important difference is that SARS-CoV-1 was predominantly spread by those with, often severe, symptoms. This feature of SARS-CoV-1 made it possible to identify who was infectious and isolate them and thus shorten the length of time individuals were infectious, driving R below 1 and ending the nascent pandemic [1]. By contrast, SARS-CoV-2 can be transmitted by presymptomatic, asymptomatic, and mildly symptomatic hosts, while still frequently causing death and severe disease, making it both difficult to contain and a significant health burden [10]. MERS-CoV, on the other hand, does not seem to have quite the right combination of characteristics to maintain large epidemics, yet frequent introductions from zoonotic hosts have continued to occur since it was first identified. Many of these MERS-CoV spillovers have been followed by human-to-human transmission, opening the way to selective pressure for this virus to adapt for efficient human spread, which raises the question, why has MERS-CoV never evolved into a pandemic pathogen?

MERS-CoV has never spread extensively beyond the Middle East, barring one nosocomial (hospital) outbreak in South Korea seeded by a returning traveler [11]. There have been sporadic cases elsewhere, including North America [12], the United Kingdom [13], France [14], and Thailand [15], but only with occasional onward transmission, usually within households [2]. Accordingly, estimates of R are often close to or below 1 [16], although there have been periods where it seemed the virus had crossed this threshold [17].

The multiple chains of transmission that have occurred since MERS-CoV emerged should have provided ample opportunity for mutations that affect the traits that make up R (transmissibility and duration of infection) to push R higher and initiate global spread. This would then increase opportunities for further response to selection, as we have seen with the emergence of improved receptor binding for SARS-CoV-1 [18] and increasingly transmissible variants of SARS-CoV-2 [19]. That this has not occurred is particularly surprising as evidence suggests that rapid evolutionary change is possible in MERS-CoV and other coronaviruses, including recombination within and between reservoir species [20] and evolution in response to immune pressure (i.e., antigenic drift) [21]. Experience shows that when mutations in MERS-CoV do occur, they can spread rapidly, as shown by the spread of 2 mutations in the spike protein during the outbreak in South Korea [22].

Understanding why MERS-CoV has failed to respond to selection and become a pandemic pathogen when SARS-CoV-2 has been so successful may provide fundamental insights into what makes one potentially emergent pathogen a pandemic threat and another not. It might also shed light on the relative importance of acquisition of critical features prior to spread within human populations (“preadaptation”) compared to acquisition during early spread. In this Unsolved Mystery, we explore the key questions and nascent evidence as to what differences between MERS-CoV, SARS-CoV-1, and SARS-CoV-2 may have driven their different fates and what research is needed to use this contrast to better understand what makes a pathogen pandemic.

### Does the role of the zoonotic host shape the prospects for pandemic emergence?

The features of the virus at the time of spillover will shape the prospects of pandemic spread and are likely to hinge on the characteristics and ongoing role of the animal reservoir (Table 1). By the time SARS-CoV-1 and SARS-CoV-2 were detected as epidemic pathogens in humans, their zoonotic reservoir had no appreciable role in their epidemiology, whereas for MERS-CoV, camels, the virus’s zoonotic host, have continued to have a role in sparking intermittent outbreaks [3]. Hence, for MERS-CoV, we may be seeing a different phase of the emergence process, where the selective pressures affecting most of the viral population are not necessarily moving viral evolution toward efficient spread in humans, whereas for the SARS viruses, we are dealing with already “emerged” pathogens (where the viral population is or was predominantly circulating in humans).

**Fig 1:**
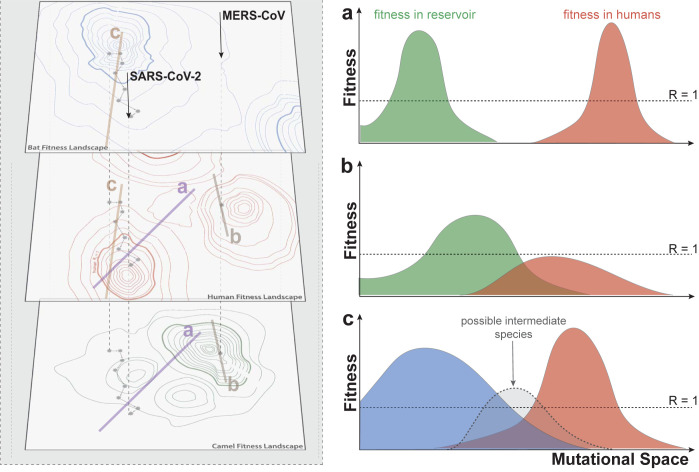
Interaction of fitness landscapes across species. Viruses can be conceptualized as inhabiting a point in a multidimensional space dictated by their genetics, where each point corresponds to some fitness (i.e., an effective reproductive number, R) in every potential host species. The relationship between these fitness landscapes dictates whether the random evolutionary walk or response to selection of a virus could lead to emergence in humans (or other species) The left panel shows a hypothetical fitness landscape where contour lines indicate fitness corresponding to a two-dimensional location (thicker contours denote the threshold of R = 1). The right panel shows slices through this hypothetical landscape at the denoted positions (lines and letters on the left-hand panel). The ability of a virus to invade a new host is dictated by its relative fitness at different points on this (high dimensional) landscape. Any evolutionary path in this landscape that must traverse an area of low fitness in both humans and the zoonotic reservoir (as in slice **a**) cannot plausibly result in emergence. Such a valley may separate the camel-adapted state of MERS-CoV with one of efficient transmission in humans. The MERS-CoV experience is, however, also consistent with there being an area of the fitness landscape that supports both efficient transmission in camels and transmission efficiency in humans just below the critical threshold (as illustrated in slice **b**), but a path between this area of subcritical fitness and one of efficient human transmission traverses a valley. In cases where R is above or marginally below 1 in the zoonotic host, and the corresponding fitness in humans is near 1, with a clear path to efficient human transmission without traversing a fitness valley (as illustrated in slice **c**), emergence can occur. This process can be aided by an intermediate host that can accommodate a virus with characteristics that would lead to efficient transmission in both the human and the primary zoonotic host, even if no area of mutual fitness exists. SARS-CoV-2 likely traversed such an area. Some hosts or host complexes may sustain viruses across a large part of mutational space, if there are many “solutions” to achieving high fitness, and/or a high recombination rate among viruses, a feature suggested in particular for bat populations [25,59]. The resulting wide fitness peak might be particularly likely to overlap with areas in mutational space where R > 1 in humans, even without an intermediate host species, as shown in slice **c**. MERS-CoV, Middle East Respiratory Syndrome Coronavirus; SARS-CoV-2, Severe Acute Respiratory Syndrome Coronavirus 2.

The successful spread of SARS-CoV-2 within human populations, and to a lesser extent that of SARS-CoV-1, may be in part attributed to fortuitous acquisition of the “right traits” while still circulating in the zoonotic reservoir, prior to emergence in the human population. This conjunction of events can be conceptualized in terms of fitness landscapes across different hosts (Fig 1). Specific characteristics that may have had a role in such “preadaptation” can be pinpointed. For example, regions of an RNA sequence in which a cytosine nucleotide is followed by a guanine nucleotide (CpG) are known to elicit strong innate immune responses [55] and are frequent in MERS-CoV, yet SARS-CoV-2 emerged with lower CpG ratios than MERS-CoV, perhaps as a result of selection in reservoir hosts [56]. Lower CpG ratios may then have made it easier for SARS-CoV-2 to elude human innate immunity. The emergence of these and other traits conducive to human spread (e.g., efficient binding of spike to human receptors [25], possession of a furin cleavage site on the spike protein that enables cleavage of the spike protein into subunits allowing efficient fusion of cellular and viral membranes [57], and other features that enhance evasion of innate immunity [56]) may have occurred by chance in bats or been accelerated by adaptation to intermediate hosts [58] (e.g., civets for SARS-CoV-1). Indeed, bats have been suggested to be hosts with unique potential to select for viruses with human pandemic potential as a result of their within-host biology [27,28] and/or uniquely high colony densities [29]. However, for SARS-CoV-1 and SARS-CoV-2, these alternative hosts ceased to have an important role once substantial human-to-human transmission was underway.

**Table 1:** Comparison of features of the 3 coronaviruses.

Characteristic	SARS-CoV-1	SARS-CoV-2	MERS-CoV
*Virus biology*			
Host range of the progenitor virus for the human pandemic	Bats and possibly civet cats [23–25]	Bats and possible intermediate hosts [26,27]	Camels (originating in bats) [28–30]
Receptor	ACE2 [31]	ACE2 [26]	DPP4 [32]
Receptor distribution (virulence)	Increased density higher in the respiratory tract [33,34]	Increased density higher in the respiratory tract [33,34]	Increased density lower in the respiratory tract [35]
***Human infection***			
Incubation period	4.0 days (median), 95% of cases, 1.3–12.8 days [36]	5.1 days (median), 95% of cases, 1.8–13.8 days [37]	5 days (mean), 95% of cases, 11.4 days [38]
Symptoms versus infectious period	Largely overlapping [39]	Infectiousness precedes [40,41]	Largely overlapping [42]
Proportion of presymptomatic and asymptomatic transmission	<11% [39]	>50% [40,41]	None confirmed [42]
Generation time (/serial interval)	8–12 days [42]	4–8 days [43]	7–12 days [42]
Infection fatality rate	<9.5% [44] (note: based on cases)	0.1%–1% [45] (note: based on country age distributions)	22% [46]
***Ecology and epidemiology***			
Reproduction number	2–4 [47,48]	2–4b [42,49]	<1 (0.6–0.9) [3]
Overdispersion parameter (i.e., superspreading)^a^	0.12–0.20 [50]	0.1–5.0 [50]	0.06–2.9 [50]
Exposure across the animal human interface	At least one instance, likely multiple exposures from palm civets over a limited period [51]	At least 1 primary jump (unknown context); multiple human–animal transmissions [52]; multiple likely jumps back to humans from human-seeded reservoir [53,54]	Ongoing camel-to-human transmission (at least 50 instances, likely 100s) [3]

^a^A lower number indicates more superspreading.

^b^Focusing on values from the early phase of the pandemic.

ACE2, angiotensin converting enzyme 2; DPP4, dipeptidyl peptidase 4; MERS-CoV, Middle East Respiratory Syndrome Coronavirus; SARS-CoV-1, Severe Acute Respiratory Syndrome Coronavirus; SARS-CoV-2, Severe Acute Respiratory Syndrome Coronavirus 2.

By contrast, MERS-CoV is perhaps best considered a virus of camels. Infection in camels is generally subclinical, and seroprevalence is high in adult camels (>70%), suggesting efficient transmission [60]. Limited genetic diversity of the pathogen in geographically distant populations of camels [61] suggests a relatively short time to the most recent common ancestor. However, the timing of divergence from related bat coronaviruses [62,63] and serological evidence of MERS-CoV (or related viruses) in samples from camels dating back to the 1980s [64,65] point to introduction into camels long before cases in humans were first detected. Regardless of the timeline, the overall MERS-CoV viral population is likely to be still responding to selection toward improved viral fitness in camels [66,67], a process which will not necessarily select for improved viral fitness in humans, and could even reduce the likelihood of MERS-CoV causing human epidemics (Fig 1).

The occurrence of repeated spillover from camels to humans might also provide another barrier to selection for efficient transmission of MERS-CoV relative to the very small number of spillovers of the other 2 emergent coronaviruses [68,69]. Repeated spillover could lead to an accumulation of “hidden immunity” in the individuals most likely to be zoonotically infected and thus make them even less likely to provide fertile ground for ongoing adaptation. An analysis of abattoir workers in Nigeria suggested increased T-cell immunity (although not antibodies) to MERS-CoV relative to individuals with little contact with camels [70]. Furthermore, as none of the workers showing evidence of immunity to MERS-CoV had experienced symptoms, the authors suggested that a mild variant might be circulating in local camels, a conjecture for which support is growing [71]. The immunity that these individuals had acquired from, presumably, camel-adapted variants might be a further barrier to spillover of more human-adapted strains.

Likewise, there may be observation biases that lead us to overestimate the number of opportunities MERS-CoV has had to adapt to humans compared to the ancestors of SARS-CoV-2, as we will inevitably fail to document most unsuccessful exposures. Because MERS-CoV is a known, highly pathogenic virus that causes limited outbreaks, we are likely to detect a relatively high percentage of spillover events. By contrast, other descendants of bat coronaviruses may make limited forays into human hosts and remain unseen. Indeed, serosurveys hint that, prior to the emergence of SARS-CoV-1 and SARS-CoV-2, in certain parts of the world, exposure to antigenically similar viruses was quite frequent [4,5,72–74]. Hence, these ancestral viruses may have had numerous failed opportunities to adapt to human spread before the critical spillover event occurred (i.e., the successful strains we see are just the result of many human exposures in which the right combination of preadaptation and in-human mutation were not present). Broad studies of prepandemic specimens with specific serological assays (e.g., neutralization tests) may help to better characterize the size of these hidden emergence opportunities and understand how the risk from these unseen events balances against the known threat from MERS-CoV, as well as whether the key limiting process might, for many viruses, indeed be exposure, rather than acquisition of the right traits (“preadaptation”).

Nevertheless, traits such as the CpG ratio do seem ripe for selection in MERS-CoV and, indeed, have repeatedly been shown to respond to selection over the course of the SARS-CoV-2 pandemic [55], so the question remains of why a response to selective pressure in human hosts has not occurred following multiple spillover events from camels.

### Does within-host biology, replication, and pathogenesis in humans enable or constrain the effect of selection toward spread?

Although conditions in the zoonotic reservoir may not have furnished MERS-CoV with the traits necessary for immediate spread within human populations, some human-to-human transmission of MERS-CoV can occur. This means that there is the opportunity for further selection and adaptation to human-to-human spread during the phase of short chains of human-to-human transmission. Why has this adaptation not happened? The answer may lie in the nature of the within-host biology of MERS-CoV compared to its relatives. Two broad aspects of within-host biology could potentially have a key role: rates of viral growth and how viral tropism (i.e., the cells the virus tends to infect) interacts with pathogenesis and transmission.

Just as R can be used to describe spread across populations, the within-host R, or the number of newly infected cells caused by an infected cell within the host, can help describe the within-host dynamics of a virus [75]. The within-host R of MERS-CoV at symptom onset is low relative to that of SARS-CoV-1 and SARS-CoV-2 [76], with values around 1.5 compared with values of >4 for the other 2 viruses; hence, MERS-CoV reaches peak viral load more slowly than the SARS viruses [76]. This slower rate of viral growth is likely to reduce transmission overall relative to SARS-CoV-2 and SARS-CoV-1 but may also have other effects on the epidemiology. One particularly important consequence is that it may lead to less presymptomatic spread in MERS-CoV. SARS-CoV-2 replicates rapidly early in the course of infection, and, not inconsequentially, it has been estimated that as much as 50% of transmission occurs before symptom onset [77], an important barrier to virus control [39]. SARS-CoV-1 seems to occupy a middle ground, with faster viral growth than MERS-CoV but not the same frequency of high presymptomatic viral loads as SARS-CoV-2 (Table 1), producing low enough levels of invisible spread that it can be contained (severity may also have a role, as detailed below). However, the mapping between symptoms and within-host viral growth remains unclear. The possible determinants of slower within-host growth of MERS-CoV include a range of features, such as less efficient evasion of innate immunity [56], potentially via less efficient disruption of innate immune signaling, a process involving many coronavirus proteins beyond the spike protein [78–80], or other features resulting in lower abundance of virus generated within each infected cell (e.g., associated with features of the viral replication machinery [79]). Comparisons of the genetic characteristics of SARS-CoV-2 variants [81] and MERS-CoV strains [82], alongside sequence analyses [79], competition-based assays and structural biophysics [83–85], and cell line [78,86] and experimental infections [78] are beginning to yield insights into the functionality of a range of coronavirus proteins and their intersection with host immunity. However, much remains to be done to fully understand the drivers of differences between the within-host biology of MERS-CoV and SARS-CoV-2. The degree to which the unusually vast host range of SARS-CoV-2 is a feature of its broad capacity to neutralize innate immunity is a particularly interesting question.

The relationship between the growth of the within-host viral population and transmission is complicated by where in the host body that growth is occurring. The location of the cells infected can both impact disease severity and ease of transmission. Like many other coronaviruses, MERS-CoV uses a ubiquitous cell surface receptor to gain access to cells, specifically dipeptidyl peptidase 4 (DPP4), a protease found across vertebrates [87]. While this ubiquity may facilitate cross-species transmission (noting that compensatory mutations in the spike are likely to be required to use the receptor in a new host species [86]), the distribution of DPP4 in the human respiratory tract may present barriers to pandemic emergence. DPP4 is less common in cells of the upper respiratory tract of humans [35,88], the location most favorable to onward transmission [89], than in the lower respiratory tract, making infection less likely; whereas angiotensin converting enzyme 2 (ACE2), the receptor used by SARS-CoV-1 and SARS-CoV-2, is common in the upper respiratory tract [90]. In addition, the concentration of infection in the lower respiratory tract caused by the location of DPP4 leads to more severe disease. This increased disease severity may limit transmission because if the virus quickly kills or incapacitates the host, that host will be unable to circulate in the population and spread the infection. Most MERS-CoV replication occurs in the lower respiratory tract [91], which likely contributes to its high mortality (an estimated 22% of humans infected with MERS-CoV die as a result of the infection [46]). By contrast, reliance on DPP4 might be advantageous for the virus in reservoir species. Indeed, camels have higher densities of this receptor in the upper respiratory tract, which is favorable to transmission [35], whereas in some bats, expression is dominant in the intestine [92], making MERS-CoV likely a relatively mild but efficiently transmitted fecal–oral pathogen in bats. Of course, cellular tropism is not necessarily a fixed property of MERS-CoV, and there is precedent for change in cellular tropism among the coronaviruses. For example, a deletion in the spike protein seems to have allowed a porcine coronavirus to shift from being an occasionally severe enteric infection (transmissible gastroenteritis coronavirus) into a mild respiratory one (porcine respiratory coronavirus) [93], and SARS-CoV-2 seems to be able to infect cells that do not produce ACE2 [94]. However, access to such a novel part of mutational space might require crossing a vast valley in the fitness landscape (Fig 1A). The Omicron variant of SARS-CoV-2 hints at how subtle changes in receptor biology can shape changes in cellular tropism: This variant relies less on transmembrane protease serine 2 (TMPRSS2) for the spike cleavage normally necessary for entering cells, instead being engulfed into endosomes. This change may have allowed Omicron to shift its tropism away from lungs and other organs that have high expression of TMPRSS2 toward the nasal passages, where cells express ACE2 but not TMPRSS2 [95].

Despite using ACE2, with its more propitious distribution across the respiratory system, the spread of SARS-CoV-1 was also impeded by high virulence, whereas SARS-CoV-2 was not (although it still has high rates of mortality compared with established human coronaviruses). The details of the kinetics of infection described above are likely to govern this difference, such as rapid early within-host cell-to-cell spread of SARS-CoV-2 [76] enabling presymptomatic transmission [77], and higher peak SARS-CoV-1 viral titers [76] potentially driving more severe outcomes. Finally, moving beyond these within-host features, considering the prospects for MERS-CoV responding to selection to achieve efficient human spread requires considering potential barriers associated with human-to-human transmission.

### Do characteristics of the transmission process limit prospects for further response to selection?

Beyond barriers associated with the biology of within-host replication and the role of the reservoir, there may also be barriers to adaptation that emerge from the details of host-to-host transmission and the overall epidemiological process. These include tradeoffs between viral fitness for within-host replication versus transmission between hosts, and the propensity for chains of transmission in humans to go extinct due to a reliance on so-called “superspreader” events.

One barrier to the establishment of mutations that increase the efficiency of viral transmission emerges from the fact that mutations that improve viral fitness within hosts (where the most direct evolutionary pressure occurs) are not necessarily beneficial for spread between hosts.

Even if an adaptation that is advantageous for population-level spread has a neutral effect on within-host dynamics, the existence of “transmission bottlenecks” may make it unlikely to be passed on. These bottlenecks occur because of the very small number of virions that are actually transmitted between hosts [96]. For SARS-CoV-2, only 1 to 8 virions, out of billions in an infected host, are estimated to be transmitted in an infection event [97]. This small probability of any individual virion being transmitted may blunt the impact of selective advantage for between-host transmission and has been invoked to explain the relatively slow pace of evolution of SARS-CoV-2 over the course of 2020 [97]. Indeed, for SARS-CoV-2, emergence of variants of concern with greater fitness occurred in the context of hundreds of millions of people having been infected worldwide, giving ample opportunities for advantageous mutations to pass these bottlenecks and establish themselves—SARS-CoV-1 and MERS-CoV have both had fewer opportunities by several orders of magnitude (i.e., the number of SARS-CoV-2 transmission events is in the billions versus numbers in the thousands for MERS-CoV and SARS-CoV-1). This problem is made worse for the virus when there is poor alignment between mutations that are advantageous within hosts and those that are advantageous for transmission (an example of epistasis) and if mutations emerge relatively slowly, as is the case for coronaviruses [98]. Strong purifying within-host selection [97] may mean that mutations advantageous for between-host spread might be removed at the phase of within-host growth. This does not seem to be the case for critical mutations in the spike protein of SARS-CoV-2 [19], where changes that enhance both within-host viral population growth and immune escape (which would aid epidemic spread) have emerged. What remains unclear is whether there are features of SARS-CoV-1 or MERS-CoV that make such jointly beneficial mutations less likely. Even with multiple opportunities for adaptation to human-to-human spread provided by multiple spillover events, the combination of transmission bottlenecks and within-host selection may have made it more unlikely for MERS-CoV variants to successfully traverse the bottlenecks created by the transmission process.

A specific example of the difficulties a virus might encounter in adapting toward more efficient epidemic spread comes from the advantage conferred in such spread by high levels of asymptomatic (or presymptomatic) transmission. Part of the rapid spread of SARS-CoV-2 is attributable to transmission from individuals who do not know that they are infected, i.e., who have not yet developed symptoms or never will. Despite the pathogenicity of MERS-CoV, asymptomatic infections do occur. This raises the question of why selection has not led to increases in the frequency of asymptomatic, or mildly symptomatic, MERS-CoV infections that could evade control and drive epidemics [39]. The spread of mutations that reduce receptor binding of the virus, tentatively associated with a diminished infection fatality ratio, was reported during the MERS-CoV outbreak in South Korea [22]. However, this chain of transmission did eventually die out, and it is unclear whether this reduction in morbidity and mortality was epidemiologically advantageous, or spread by chance alone [99]. If host factors dominate the pathogenic response to the virus, it may be all but impossible for the virus to adapt toward lower pathogenicity, because identical viral genomes can cause very different case outcomes, and thus the viral genome is immaterial to the outcome at the host scale. Possible reduced pathogenicity of the SARS-CoV-2 Omicron variant that emerged in late 2021 has been attributed to increased replication in cells from higher in the human respiratory tract [100], indicating that there may be room for viral evolution toward reduced pathogenicity, although it remains unclear whether the virulence of Omicron is actually reduced in immunologically naive populations.

Beyond chance events associated with transmission bottlenecks, the stochastic nature of transmission may also limit the spread of variants that increase population-level fitness. For MERS-CoV, as for many other coronaviruses, the number of new infections per infected individual can be highly variable, with some individuals infecting many others (up to 150 in the outbreak in South Korea) and others infecting none [101]. Such chance variability in infectivity among individuals will tend to reduce the probability that an adaptive variant becomes established because, by chance, adaptive variants might arise or end up in individuals who result in no onward transmission [102,103]. Although SARS-CoV-2 shows striking overdispersion [104], higher overall transmissibility (R) than MERS-CoV is likely to make this less of a barrier to the spread of variants that increase fitness.

### Comparing MERS-CoV and SARS-CoV-2 as a pathway to understanding the pandemic potential of emerging pathogens

A challenge in the study of emerging pathogens is disentangling the properties of a virus that will allow it to spread in human populations from the processes that give the virus the opportunity to infect and potentially respond to selection to adapt to human hosts. Because MERS-CoV spillover events are identified with regularity, studying the differences between MERS-CoV and SARS-CoV-2 may help us to disentangle those properties. There may be coronaviruses in bat populations that, given enough opportunities to infect human hosts, could rapidly spread and become pandemic (either as a result of being “preadapted” or as a result of responding to selection during early transmission events, or both), whereas others cannot. Although specificities of the coronavirus group may complicate generalization, comparison of failed and successful emergence among the coronaviruses may provide a lens on common features of pathogens with pandemic potential. As MERS-CoV seems to be, so far, on the “cannot adapt in response to selection” side of this equation, understanding why can help us to better understand that larger pool of viruses.

This comparison may be a more fruitful path toward understanding emergence than research centered around pinpointing singular spillover events or gain of function experiments that attempt to create laboratory conditions that drive competent viruses to evolve toward increased potential for human-to-human transmission (as opposed to those focused on understanding mutation in particular proteins under controlled conditions). The former may put too much stock in events that are mostly the result of random chance and relies on identifying spillover events that may not be observable, whereas the latter can be dangerous [105] and create a myopic focus on singular mutations. A fruitful direction of research is to combine the wealth of advances in understanding the molecular mechanisms underpinning differences between MERS-CoV and SARS-CoV-2 (from genetic, structural, cell line, and model organism research) with consideration of the tools of disease ecology, computational simulation, and fundamental biology. This perspective can help researchers to identify what general foundational data should be expanded, what measurements taken across host and pathogen species are likely to shed light on relevant virus–host interactions, and what dimensions of characterization of ecological systems will be most fruitful for defining pathogen pandemic potential. We detail each in turn.

### Improving foundational data

Understanding the extent and impact of exposure to the vast population of zoonotic coronaviruses would benefit from large-scale serological studies that combine screening using broad serological assays [106,107] with more specific functional assays (e.g., neutralization tests) and assessments of cellular immunity [108]. This could reveal an important missing piece of the puzzle: the frequency of spillover for largely asymptomatic or mild viruses. Likewise, questions about how transmission interacts with pathogenesis for SARS-CoV-2 and MERS-CoV, and other viral properties, would benefit from expanded sequencing efforts in which sequence data are accompanied by data on symptoms, including their time course.

### Better understanding of host–virus interactions

The host and viral properties that lead to differences in the rate of growth of viral populations (and their relations to symptoms) in MERS-CoV and SARS-CoV-2 point to factors that might, if measured, indicate potential original or intermediate hosts of pandemic-potential pathogens. For example, building an atlas of the location, abundance and cross-species diversity of possible viral receptors through the host respiratory tract, and potentially other parts of the host body [35,92] could shed light on receptors (and hosts) likely to be associated with transmission. The distribution of such receptors in our own bodies might point to receptors likely to be associated with virulence. Such data would allow us to answer questions such as whether camels have an unusual distribution of DPP4; how conserved the locations and structures of known coronavirus receptors are across hosts; and whether the location and structure of DPP4 is less conserved among mammalian hosts than ACE2, such that the dependence of MERS-CoV on DPP4 restricts it to a narrower host breadth [109]. Similar measurements on the virus side could indicate the breadth of the range of receptor variation that is still compatible with cellular attachment through probable mutational pathways, for MERS-CoV in particular [86,109], and coronaviruses in general. Given the full gamut of approaches available from in silico to in vitro expression of synthesized host receptor proteins and viral spike proteins (ranging across both existing and potential sequences) and experimental infections of potential host animals, there is potential to construct a more complete picture of this interaction space. Understanding the range and geography of host–virus pairs that suggest pandemic potential will also shed light on the importance of human exposure to such viruses (which could be rare relative to their occurrence in host populations). Although much work to date has focused on spike receptor binding, finding tractable ways of probing functionality and relevance of other open reading frames in a generalized multihost, multipathogen way is also an important question. It would be of great interest to establish the relative importance of spike receptor configuration in overall host–virus interactions compared with all other viral genes, especially in the context of curating the direction of efforts expended in characterizing pathogens with pandemic potential.

### Modeling and characterizing ecological systems

To better link host-level factors to population-level factors, we should hone our computational approaches, focusing on the observed differences between MERS-CoV, SARS-CoV-1, and SARS-CoV-2 in terms of receptor binding, immune response, and resulting impact on transmission, as opposed to modeling abstract virulence versus transmission tradeoffs. Such modeling should include exploration of other critical transmission processes that may impact emergence, including the relationship between overdispersion, mean R, and probability of emergence (here too, focusing on where we have real data). Finally, we should look for evidence that the processes models suggest might be important are actually occurring in the real-world disease systems. For example, the suggestion that MERS-CoV is still responding to selection in camels [66,67] makes it of interest to establish whether this might be at the expense of efficient spread in humans; similar understanding of selection pressures on SARS-CoV-2 during spillback outbreaks in deer [110] and other hosts (e.g., HCoV-229E may have spread from human to alpaca populations decades ago [111]) would also be very informative.

## Concluding remarks

Although progress is already being made on many of the threads discussed above, such as expansion of our knowledge of receptor distribution and characteristics [109], broader surveillance of exposure or infection among potential reservoir hosts [25], and better understanding of unusual features of immune responses of common reservoir hosts such as bats [112] or aspects of host competence to shared pathogens [113], much remains to be done to understand how the genetic characteristics of a virus translate into the prospect of it driving the next pandemic. Further building on the research threads described here may help us to better understand the important contrasts in the pathogen genetics and ecology of MERS-CoV and the SARS viruses that have led to the pandemic success of SARS-CoV-2 and the failure of the others and thereby illuminate the larger space of what makes pathogens successfully jump to human hosts (Fig 1). MERS-CoV is of particular interest because it is unique among potentially emergent zoonotic respiratory pathogens, in that the process of exposure is relatively well observed, and the virus is sufficiently well adapted to humans to cause significant outbreaks, but it does not, as yet, have the features needed to cause a pandemic. By contrast, avian influenza A viruses such as H5N1 and H7N9, although also having relatively well-documented exposure rates, have rarely caused onward transmission and never for more than 1 to 2 generations, despite many hundreds of documented spillover events (862 and 1,565, respectively) [114–116]. Deep study of “model systems” has repeatedly proven fruitful in the study of infectious diseases (much of our fundamental understanding of disease dynamics stems from detailed study of measles), and by focusing on the contrast of MERS-CoV and SARS-CoV-2, we may develop theories and gain insights that we can eventually apply to the larger space of potentially emergent pathogens and bring us closer to the goal of actually being able to foresee a pandemic before it happens.

## Abbreviations

ACE2: angiotensin converting enzyme 2
DPP4: dipeptidyl peptidase 4
MERS-CoV: Middle East Respiratory Syndrome Coronavirus
SARS-CoV-1: Severe Acute Respiratory Syndrome Coronavirus
SARS-CoV-2: Severe Acute Respiratory Syndrome Coronavirus 2
